# Automatic detection of fungiform papillae on the human tongue via Convolutional Neural Networks and identification of the best performing model

**DOI:** 10.1016/j.csbj.2025.05.014

**Published:** 2025-05-14

**Authors:** Lala Chaimae Naciri, Raffaella Fiamma Cabini, Melania Melis, Roberto Crnjar, Diego Ulisse Pizzagalli, Iole Tomassini Barbarossa

**Affiliations:** a Department of Biomedical Sciences, University of Cagliari, Monserrato, CA 09042, Italy; b Euler Institute, Università della Svizzera Italiana, Lugano, Switzerland; c International Center for Advanced Computing in Medicine, University of Pavia, Italy

**Keywords:** Taste perception, Fungiform Papillae (FPs), Convolutional Neural Networks

## Abstract

Fungiform papillae (FPs) are fundamental for taste perception, as they contain the taste sensory cells responsible for detecting taste stimuli. Variations in the number and functionality of FPs among individuals lead to differences in taste perception, impacting the ability to identify nutrient-rich foods, health, and the joy of consuming tasty foods. Detecting FPs is a complex and time-consuming task, and there is no consensus on manual and automated methods for their identification and analysis. Objectives: This work aimed to provide an efficient, reliable, and automatic method for FP identification on the tongue, considering the physiological variations in morphology and distribution among subjects. Methods: We used three different Convolutional Neural Networks as a regression task on 175 images of the tongue, the Classic U-Net, the MultiResUNet, and the Optimized U-Net, designed to enhance the performance also when it must identify FPs in challenging input images. Results: The Optimized U-Net showed the best performance by achieving the lowest errors and the highest similarity between Ground Truths and prediction values, and the more balanced detection of True Positives, Untrue Negatives, and Untrue Positives. Conclusions: Our results show that the Optimized U-Net achieved the highest stability, accuracy, and robustness in learning and prediction of FPs with challenging morphologies. The ability to automatically detect FPs has important implications for understanding individual differences in taste perception, which could eventually help in diagnosing taste disorders or guiding personalized nutrition plans.

## Graphical Abstract

**Figure:**
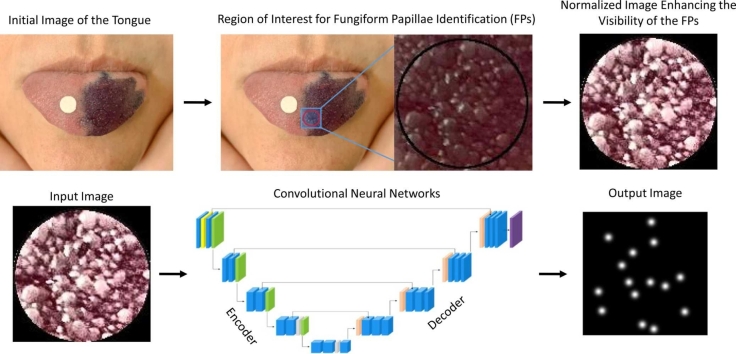

## 1 Introduction

Taste sensitivity is essential for human survival, well-being, and overall health. It serves as a last checkpoint for food acceptance or rejection behavior and helps organisms differentiate between nutrient-rich and toxic substances [1], [2], [3]. Interestingly, taste sensitivity shows considerable individual physiological variability among healthy humans, with direct consequences on food preferences, eating habits, and long-term health outcomes, such as the risk of obesity, diabetes, or malnutrition [4], [5], [6], [7], [8]. In addition, taste sensitivity can be influenced by various external factors [9], [10], [11], [12] or pharmacological interventions, such as many antibiotics and sleep aids [13], [14], [15], even though they do not change the number of papillae. Many pathological disorders [16], such as neurological diseases [17], [18], [19], autoimmune disorders [12], [20], infections [21], or metabolic disorders [22] may also cause a decrease in taste sensitivity called ‘hypogeusia’ or a complete taste loss ‘ageusia’.

These taste dysfunctions can negatively impact the quality of life, decreasing food enjoyment with negative consequences, which could determine excessive weight loss or gain leading to malnutrition [23]. Taste perception in humans begins with the activation of the taste cells located in taste buds [24], which are organized into different types of papillae mostly distributed across the upper surface of the tongue [25]. Three different types of taste papillae contain taste buds, each with specific morphological features and locations on the tongue: Circumvallate papillae which are located in the posterior one-third of the tongue, Foliate papillae on the lateral side, and fungiform papillae (FPs), which are mushroom-shaped structures topographically arranged in the anterior two-thirds of the tongue, interspersed among filiform papillae that do not contain taste buds [26].

Most studies on taste perception have focused on FPs because they are the most numerous and contain the most taste receptors, making them crucial for identifying the five primary tastes: sweet, salty, sour, bitter, and umami, as well as a potential sixth taste quality which may include fatty acids [27]. The variability in the number and structure of FPs among individuals has long been a topic of interest in sensory and nutritional sciences [28]. However, identifying FPs on the tongue is a difficult and time-consuming task, and there is no consensus on manual and automated identification methods. This highlights the need for more reliable methods for the identification of FPs and their quantification and analysis [29].

Traditionally, the identification and counting of FPs have been performed manually using the visual observation of the tongue's surface. This process involves the use of blue alimentary dye, which colors the filiform papillae and the FPs differently, highlighting their mushroom-like shape [26], [29], [30]. This manual technique is considered a gold standard in taste research [29]. However, it is time-consuming and implies highly subjective evaluations that produce errors of quantification, especially when applied to large datasets and large-scale studies where speed, accuracy, and reproducibility are critical. To address these limitations, automated methods for identifying and counting FPs have been explored, offering the potential to standardize the process and enhance efficiency and reliability [31], [32], [33], [34]. Among these, Deep Learning (DL) approaches based on Convolutional Neural Networks have shown great promise in the analysis of biomedical images [35], [36]. In particular, the U-Net architecture has been widely applied in medical imaging for tasks such as tumor detection, organ segmentation, and cell counting, demonstrating robustness across various applications [37], [38]. Recent studies [31], have demonstrated the effectiveness of U-Net in segmenting FPs from tongue images, achieving strong correlations with manual counting methods. However, in this work, a limited dataset has been used to train the model. Only 29 tongue images were used for the training and validation of the papillae segmentation model. Despite the potential, the use of these methods is still limited outside of specialized users, mainly because there are no open-source versions easily accessible to end users.

This work aimed to automatically identify the FPs on the tongue images of 175 healthy subjects, considering the natural variations in morphology and distribution among subjects. Our goal was to overcome the challenges and limitations associated with manual counting and provide an automatic and standardized method that is efficient and reliable in FP identification since it can generate a probability map as a regression task instead of a previously used binary mask [31]. To this aim, we used and compared three different Convolutional Neural Networks (Classic U-Net [37], MultiResUNet [39], [40], and an optimized version of U-Net that we designed to enhance the performance in detecting FPs even when it has to identify FPs in challenging input images characterized by different density and morphology. Our method can be used by all experts working on taste physiology, making the script available and open source on GitHub.

## 2 Materials and methods

### 2.1 FPs identification and measurements

Identification and measurements of FPs were performed on one hundred and seventy-five young, healthy Caucasian subjects (62 male, 113 female) from Sardinia, Italy, recruited at the local university. The mean age was 24.77 ± 4.74 y, ranging from 18 to 42 y; 22 subjects were smokers and 153 were non-smokers. A structured history was taken for each subject. They had a normal body mass index (BMI) ranging from 20.2 to 25.2 kg/m^2^. The exclusion criteria included: major systemic diseases, otolaryngology disorders, neurodegenerative diseases, use of drugs interfering with taste or smell, pregnancy or lactation, and food allergies. Taste and olfactory impairment were ruled out by using the Taste Strips and Sniffin’ Sticks test (Burghart Messtechnik, Wedel, Germany). All subjects provided a signed informed consent form before being enrolled in the study. The study was conducted according to the guidelines of the Declaration of Helsinki and approved by the Ethical Committee of the University Hospital of Cagliari (protocol code: 451/09; date of approval: 5/2016).

A standardized procedure was used to identify the FPs on the tongue of these subjects [29]. Briefly, the tip of the anterior tongue surface was dried using filter paper and stained with a blue food dye (E133, Modecor Italiana, Italy) on the left side of the midline of the tongue. Multiple photographs (3−10) of the stained area were taken using a Canon EOS D400 (10 megapixels) camera equipped with an EFS 55–250 mm lens. After transferring the images to a computer, they were analyzed by the zoom function in GIMP Software. The FPs were identified in a 6 mm diameter circle of stained and dried area according to Melis [29]. This circular area provides reliable measurements of the density of FPs in high correlation with their total number on the tongue [30]. The FPs were identified by their mushroom shape and lighter staining, which allowed them to be distinguished from the darker-stained filiform papillae. The number of FPs in the stained area of 6 mm diameter was counted by three trained observers, who independently evaluated the image. The final measurements were based on the consensus of all observers.

### 2.2 Deep Learning methods

**Fig. 1:**
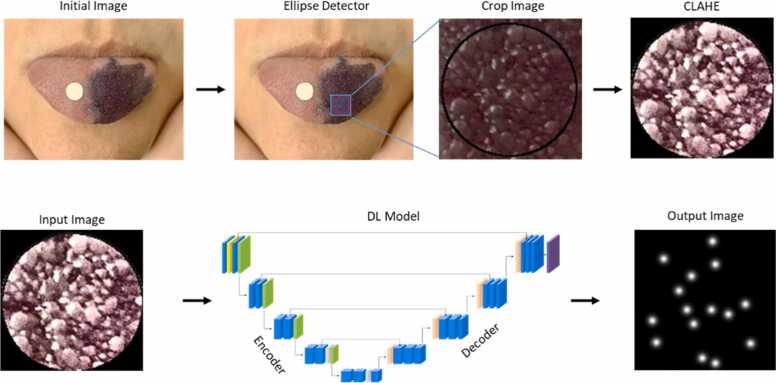
Workflow of the automatic identification of the FPs by Deep Learning architectures. Initial Image, the best image of each subject; Ellipse Detector shows the circular region where the FP measurements are performed; Crop Image, the previous image cropped after applying an ellipse detector; Contrast Limited Adaptive Histogram Equalization (CLAHE) image after normalization and enhancing the visibility of the FPs.

To automatically identify the FPs on the tongue images of 175 healthy subjects, considering natural variations in morphology and distribution among subjects, we used and compared three different Convolutional Neural Networks (Classic U-Net, MultiResUNet, and an Optimized U-Net). Our workflow is illustrated in Fig. 1. It starts with the Initial Image of the tongue (selected as the best image of each subject). Then, it follows with a second image (Ellipse Detector) showing the circular region of interest where the FP measurements are performed. In this image, an ellipse detector was employed to identify the ellipse, which was subsequently cropped into a square centered around this ellipse (Crop Image). The Crop Image is then standardized to a uniform resolution of 250 × 250 pixels. Finally, Contrast Limited Adaptive Histogram Equalization (CLAHE) is applied for image normalization, enhancing the visibility of the FPs. The processed images and Ground Truth Images, generated as described below, are then fed to the end-to-end DL models, which generate output images featuring bright spots at the center of the FPs.

#### 2.2.1 Generation of ground truth images

**Fig. 2:**
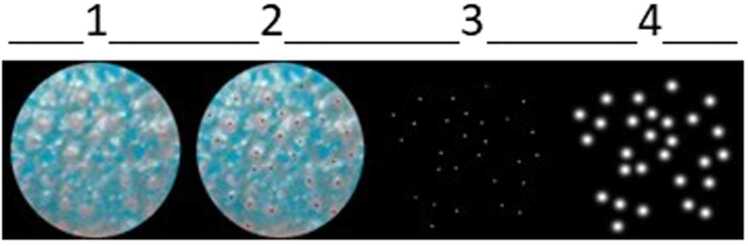
Image processing pipeline for creating the Ground Truth images. Three steps were followed: 1 → 2, manual identification of all FPs using GIMP Software; 2 → 3, isolation of the points indicating each FP position; 3 → 4, creation of Ground Truth Image using Peak Local Maxima Detection and Gaussian kernels.

Three steps were followed to generate the Ground Truth Images needed to train the models (Fig. 2). With this pipeline, we obtained a uniform and high-quality dataset to train the three models. The three steps were:

1. The normalized images were manually annotated by marking the position of all FPs using GIMP Software.
2. Isolation of the points indicating each FP position.
3. Creation of Ground Truth Image using Peak Local Maxima Detection [41] and Gaussian kernels [42].

#### 2.2.2 Deep learning architectures for FP identifications

The three architectures (Classic U-Net, MultiResUNet, and an Optimized U-Net) have been used as a regression task, with a high probability of bright spots at the center of the FPs that gradually decreases toward the borders. To this end, we used a linear activation function in the final layer of all architectures [38].

Classic U-Net [37]: a standard U-Net architecture, composed of an encoder-decoder structure, each composed of 4 blocks of 2D convolutional layers. The encoder progressively downsamples the input image to extract relevant features, while the decoder upsamples the feature maps from the encoder to gradually reconstruct the original image resolution. A middle block of convolutional layers connects the encoder and the decoder. The encoder progressively doubles the number of the convolutional filters in each block, starting with 64 filters in the first block and reaching 1024 filters in the middle block.

MultiResUNet [39]: an advanced U-Net architecture incorporating multi-resolution convolutional blocks within the encoder and decoder. These blocks allow the network to extract features at different scales, improving the model's ability to capture fine-grained details at various resolutions.

Optimized U-Net: The improvements made in the Classic U-Net to enhance the performance in the detection of FPs were: reducing the number of filters in the encoder and decoder blocks to 64 convolutional filters in the first two blocks, 128 in the third, 256 in the fourth, and 512 in the middle block, incorporating Batch Normalization layers after each convolution to stabilize and speed up training, and adding Dropout layers to mitigate overfitting.

#### 2.2.3 Training deep learning models

After processing the data, to train and validate the models we used the K-fold cross-validation strategy [43]. The k-fold cross-validation is a technique for the statistical evaluation of DL models that trains and assesses them on data subsets. This procedure was repeated five times (k = 5): each subset was randomly used once as a validation set and the remaining four for training. At each of these five times, the model runs for 500 epochs with a batch size of 2. This approach provided robust model evaluation by assessing performance across different training-validation splits, enhancing the reliability of the results.

In addition, we used the Mean Squared Error (MSE) as the loss function, and the Mean Absolute Error (MAE) as the error quantification after each epoch. They were optimized with the Adam optimizer (learning rate: 1e-3). The training data were augmented with rotations, flips, and intensity adjustments to improve the model's generalization. Final performance metrics are reported as the mean and standard deviation across the five hold-out folds.

#### 2.2.4 Evaluation of results of deep learning models

To evaluate the overall performance of the three models used to predict the position of each FP we first used the Mean Absolute Error (MAE) which quantifies the average of errors between the Ground Truth and the prediction made by each model [44] after the end of training. Second, we used the Structural Similarity Index Measure (SSIM) which evaluates the overall structural similarity between the Ground Truth and the prediction [45].

In addition, we performed two deeper analyses (Dice Coefficient, DC and Local Peak Detection, LPD) to compare the prediction that the model returns with the corresponding Ground Truth. The DC calculates the degree of the overlap between predictions and the Ground Truths summing the pixels that coincide between them. DC = 0 means no overlap, DC = 1 means perfect overlap. LPD allowed us to extract the coordinates of the spots and calculate the rate of True Positive (TP), the rate of Untrue Positive (UP), and the rate of Untrue Negative (UN) with respect to the Ground Truth for all predicted FPs of each model evaluated.

Final performance metrics are reported as the mean and standard deviation across the five hold-out folds.

**Table 1:** Meaning of True Positive (TP), Untrue Positive (UP), and Untrue Negative (UN) of the prediction of FPs that the models return with the corresponding Ground Truth True image.

Model prediction	Meaning
True Positive (TP)	Correct prediction
Untrue Positive (UP)	Incorrect prediction: The model identified an FP that does not exist.
Untrue Negative (UN)	Unrecognized FPs: The model did not identify an FP that exists.

### 2.3 Statistical analysis

One-way analysis of variance (ANOVA) was used to analyze differences in mean values ± SD of the number of FPs in the Ground Truths and those predicted by each model. Post hoc comparisons were conducted with Tukey HSD test. Statistical analyses were conducted using STATISTICA for WINDOWS (version 7; StatSoft Inc, Tulsa, OK, USA). P values ≤ .05 were considered significant.

## 3 Results

**Fig. 3:**
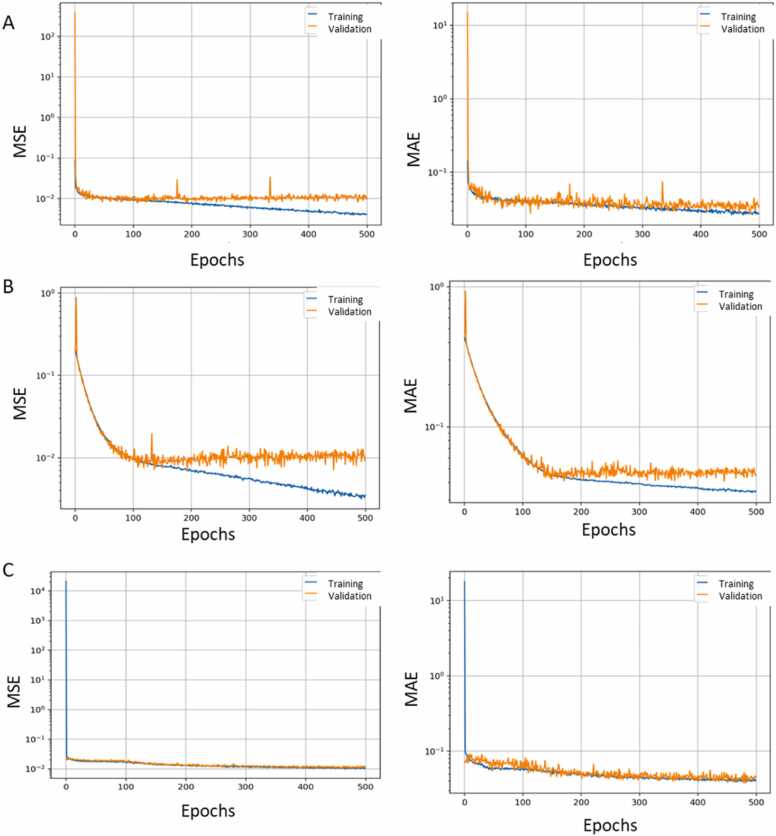
The history of the Mean Squared Error (MSE), as the loss function (left graphs), and the Mean Absolute Error (MAE), as quantification of error after each epoch (right graphs) for a Representative Fold of the Classic U-Net (A), the MultiResUNet (B), the Optimized U-Net (C). The blue curve represents training, and the orange one represents validation. The X-axis represents the number of epochs, and the Y-axis represents the logarithmic scale, which illustrates the variation of the metric values.

Fig. 3 shows the values of over 500 epochs (the history) of MSE, as the loss function (left graphs), and MAE, as the error quantification after each epoch (right graphs) monitored for a representative fold of the Classic U-Net (A), the MultiResUNet (B), the Optimized U-Net (C) to evaluate the training and validation of models. In all the models we observed a quick decrease of MSE and MAE, during the first epochs, which indicates effective early learning. Specifically, the Classic U-Net model showed higher convergence for both training and validation metrics than the MultiResUNet model which indicates better performance. The MultiResUNet model shows that the training values decrease and those of validation remain constant which suggests overfitting, and a slightly unstable training process, highlighted by the oscillations in the MSE and MAE validation values. The optimized U-Net model achieved the most balanced performance and stability because the curves show consistent trends in both training and validation, with small deviation and small stabilized MSE and MAE values.

Table 2 shows the mean values ± SD of MAE, SSIM, and DC, across the five hold-out folds, the metrics used to evaluate the overall performance of the three models to predict the position of each FP. The Optimized U-Net showed the best performance by archiving the lowest value of MAE, which quantifies the average of errors between Ground Truths and the predictions, the highest SSIM values, which evaluate the similarity between Ground Truths and predictions, and a DC value (0.712) that indicates a very good overlap between Ground Truths and predictions.

**Table 2:** Mean values ± SD of Mean Absolute Error (MAE), Structural Similarity Index Measure (SSIM), and Dice Coefficient (DC), across the five hold-out folds, for each model.

**DL Model**	**MAE ± SD**	**SSIM ± SD**	**DC ± SD**
Classic U-Net	0.036 ± 0.029	0.666 ± 0.084	0.713 ± 0.058
MultiResUNet	0.047 ± 0.002	0.258 ± 0.027	0.702 ± 0.055
Optimized U-Net	0.034 ± 0.001	0.755 ± 0.047	0.712 ± 0.033

Table 3 shows the mean values ± SD of the number of FPs in the Ground Truths and those predicted by each model. One-way ANOVA showed that the number of FPs in Ground Truths and that predicted by other models was significantly different (F_(3, 696)_ = 63.818, *p* = 0.0000). However, the post hoc comparison showed that the number of FPs predicted by the Classic U-Net was higher than that of Ground Truths or that predicted by the other two models (*p* < 0.000008; Tukey HSD test). No significant difference in the number of FPs predicted by the MultiResUNet or Optimized U-Net was found when compared to that of Ground Truths (*p* ≥ 0.827; Tukey HSD test) or between them (*p* = 0.947; Tukey HSD test).

**Table 3:** Mean values ± SD of the number of FPs in the Ground Truths and that predicted by each model.

**Ground Truths**	**Classic U-Net**	**MultiResUNet**	**Optimized U-Net**
16.96 ± 7.67	29.25 ± 13.18*	17.84 ± 8.15	18.40 ± 7.76

* Indicate significant differences compared to the other values (p < 0.000008; Tukey HSD test, after one-way ANOVA).

**Fig. 4:**
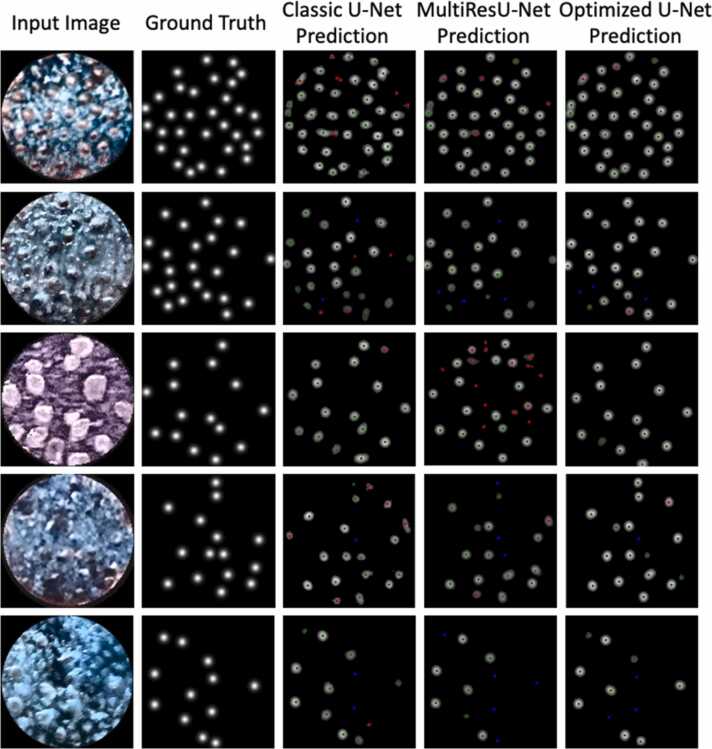
Representative examples showing the prediction of the three models the Classic U-Net, the MultiResUNet and the Optimized U-Net. Green points indicate True Positives (TPs), red points indicate Untrue Positives (UPs) and blue points indicate Untrue Negatives (UNs).

LPD analysis by extracting the coordinates of the spots in each image allowed us to calculate the rate of True Positive (TP), the rate of Untrue Positive (UP), and the rate of Untrue Negative (UN) with respect to the Ground Truth for all predicted FPs by each model. Five representative examples of the ability of each model to identify the FPs in challenging input images characterized by different FP densities and morphology are shown in Fig. 4. TPs (green points), UPs (red points), and UNs (blue points) for each model are highlighted. The predicted number of True Positives (TPs), Untrue Negatives (UNs), and Untrue Positives (UPs) calculated with respect to Ground Truth by each model for FPs in five representative examples of input images are shown in (Table 4). The Optimized U-Net showed the best balance prediction across all examples.

**Table 4:** The predicted number of True Positives (TPs), Untrue Negatives (UNs), and Untrue Positives (UPs) calculated with respect to Ground Truth by each model for FPs in five representative examples of input images.

**Example**	**Ground Truth**	**DL Model**	**TPs**	**UNs**	**UPs**
1	32	Classic U-Net	32	0	7
		MultiResUNet	32	0	4
		Optimized U-Net	32	0	2
2	24	Classic U-Net	22	2	4
		MultiResUNet	20	4	0
		Optimized U-Net	21	3	1
3	15	Classic U-Net	15	0	1
		MultiResUNet	15	0	15
		Optimized U-Net	15	0	0
4	15	Classic U-Net	14	1	5
		MultiResUNet	13	3	2
		Optimized U-Net	14	1	1
5	12	Classic U-Net	10	2	1
		MultiResUNet	7	5	0
		Optimized U-Net	9	3	0

Table 5 shows the mean values ± SD across the five hold-out folds of the number of FPs of the Ground Truths and the predicted rate of True Positives (TPs), Untrue Negatives (UNs), and Untrue Positives (UPs) calculated by each model for FPs of 175 images. The table also shows the percentages of TPs, UNs, and UPs calculated across the five hold-out folds, taking into account the real number of FPs in each fold. Specifically, the Classic U-Net model gave the highest rate of TPs and UPs and the lowest rate of UNs. The MultiResUNet gave the highest rate of UNs, the lowest rate of TPs and the lowest rate of UPs rate. Finally, the Optimized U-Net model showed intermediate values that were more equilibrated than those of other models: a slightly lower rate of TPs than that of Classic U-Net, a lower rate of UNs than that of MultiResUNet, and a lower rate of UPs than that of Classic U-Net.

**Table 5:** The mean values ± SD across the five hold-out folds of the number of FPs of the Ground Truths and the predicted rate of True Positives (TPs), Untrue Negatives (UNs), and Untrue Positives (UPs) calculated by each model for FPs of 175 images.

**Ground Truths ± SD**	**DL Model**	**TPs ± SD %**	**UNs ± SD %**	**UPs ± SD %**
592.8 ± 69.75	Classic U-Net	515.6 ± 59.98 87.06 ± 4.22	77.2 ± 26.46 12.92 ± 4.22	212 ± 130.57 37.33 ± 24.79
	MultiResUNet	444 ± 83.86 74.51 ± 6.13	148.8 ± 28.30 25.48 ± 6.13	98.4 ± 21.197 16.77 ± 4.02
	Optimized U-Net	469.4 ± 42.43 79.41 ± 3.77	123.4 ± 33.36 20.57 ± 3.77	136.8 ± 75.14 24.31 ± 15.06

The percentage of TPs (TPs/Ground Truth*100 %), that of accurate count (TPs/TPs+UPs*100 %), and complete accuracy (TP/TP+UP+UN*100 %) calculated by the three modes are shown in Table 6. Specifically, the Classic U-Net model gave the highest percentage of TPs and the lowest of accurate count and complete accuracy. The MultiResUNet gave the lowest percentage of TPs, the highest percentage of the accurate count, and an intermediate value of complete accuracy. The Optimized U-Net gave an intermediate percentage of TPs and the accurate count, while the complete accuracy was the highest value compared to the other two models.

**Table 6:** The percentage of TPs (TPs/Ground Truth*100 %), and that of accurate count (TPs/TPs+UPs*100 %), and complete accuracy (TP/TP+UP+UN*100 %) calculated by the three modes.

**DL Model**	TPs/Ground Truth* 100 %	TPs/(TPs+UPs)* 100 %	TP/(TPs+UPs+UNs)* 100 %
Classic U-Net	87.06	69.98	63.40
MultiResUNet	74.51	81.62	63.81
Optimized U-Net	79.41	76.56	63.89

## 4 Discussion

The main aim of this work was to provide an efficient, reliable, and automatic method for identifying FPs on the tongue, also considering physiological variations in morphology and distribution among subjects. To achieve this aim, we used and compared three different Convolutional Neural Networks capable of generating a probability map as a regression task, with a high probability at the center of the FPs that gradually decreases toward the borders. Our results showed that the modifications made in the Classic U-Net architecture allowed us to stabilize and speed up its training, mitigate overfitting, and improve prediction by identifying the best-performing model. The metrics used to evaluate and compare the training and validation of the models and assess their performance in learning and prediction showed that the Optimized U-Net had superior accuracy and robustness than the other two DL models. Specifically, the history of over 500 epochs of the metrics used to evaluate the training and validation of the models, MSE as a loss function, and MAE as an error quantification after each epoch, showed a rapid decrease during the first epochs, which indicates successful early learning for all models. The Classic U-Net showed a better performance than that of MultiResUNet, as shown by the higher convergence of the curves. Instead, the non-convergence and the higher oscillations of the values obtained by MultiResUNet suggest overfitting and a slightly unstable training process for this model. On the other hand, the metric curves determined with the Optimized U-Net showed consistent trends in both training and validation, with modest deviation and stable MSE and MAE values, indicating that this model achieved the most balanced performance, good stability, and no overfitting.

Our results also showed that the Classic U-Net predicted a significantly higher number of FPs than that obtained by the manual counting (Ground Truths), while the MultiResUNet or Optimized U-Net predicted a number of FPs not different from that of the manual counting. It is important to note that our approach, by identifying the number of the FPs in the small circular area (6 mm in diameter) stained with the blue food dye, provides reliable density measurements in the whole tongue. Indeed, it is known that the density measurements in this area are in high correlation with their total density on the tongue [30]. Additionally, the metrics used to evaluate the overall performance of the models showed notable differences in the learning process for identifying FPs. In particular, the Classic U-Net showed a stable performance. The values of MAE, which quantify the error after each epoch, the DC, which calculates the overlap between predictions and the Ground Truths, and the SSIM, which evaluates the overall structural similarity between the Ground Truths and the predictions, indicate a better accuracy of the Classic U-Net in predicting the FPs compared to MultiResUNet. However, the Classic U-Net showed the highest ability to identify TPs, but also the highest rate of UPs and a low rate of UNs, as well as the lowest of accurate count and complete accuracy, suggesting that it tends to confuse other types of papillae with the Fungiform ones. Hence, the high rate of TPs does not mean that it has effectively learned to recognize the morphology of FPs. These findings suggest that further refining of this model is needed to lower the rate of UPs. The MultiResUNet showed the highest MAE and the lowest SSIM and DC, which indicate a weaker performance in capturing fine details and accurately predicting the positions of the FPs compared to other models. In addition, even though MultiResUNet showed the highest accurate count and intermediate values of complete accuracy and the lowest rate of UPs, the lowest rate of TPs and the highest rate of UNs, confirm an observable difficulty in accurately detecting FP positions. The Optimized U-Net outperformed the other two models. Specifically, it achieved the lowest error (MAE), the highest similarity (SSIM), and an overlap (DC) very close to maximum, indicating the highest accuracy and precision of this model in predicting the FPs. The Optimized U-Net also showed intermediate values of TPs, UNs, UPs and accurate count, and the highest complete accuracy, indicating a more balanced detection of TPs, UNs and UPs compared to other models. Interestingly, this last value was much lower than that of the Classic U-Net, indicating fewer errors, while the UN rate was lower than that of the MultiResUNet, indicating fewer FP position misdetections. The percentage of accurate counts would seem to indicate that MultiResUNet is the most recommendable model. However, the evaluation of the performance of a model cannot be captured by a single metric, but it needs to be evaluated by looking at all metrics collectively. Therefore, although MultiResUNet showed a better accurate count (81.62 % vs 76.56 %), the Optimized U-Net showed a higher sensitivity (TPs/Ground Truth: 79.41 % vs 74.51 %), identifying approximately 5 % more FPs present in the images, and showed a slight superiority of the complete accuracy that also considers the UNs. Our results also showed that the Optimized U-Net had a higher stability, compared to MultiResUNet, with a lower SD of TPs (3.77 % vs 6.13 %) and a superior performance in case of high morphological variability, as shown in the five examples of Fig. 4 and Table 4.

Although the Optimized U-Net demonstrated the best performance among the three models, there is potential for further improvement. The procedure that uses blue food dye for coloring the tongue offers the advantage of allowing the identification of FPs by their mushroom shape and lighter staining, distinguishing them from the darker-stained filiform papillae. However, other methods that make the images sharper could be evaluated. A limitation of our method is that the tongue surface is non-flat, making it difficult to distinguish FPs for creating the Ground Truths, which were used for learning the models. Future studies would benefit from a method for flattening the tongue. Our future work should focus on reducing both the UP and UN rates by adding more images to the dataset that could further enhance the model's performance. Convolutional Neural Network architecture typically require large datasets to achieve optimal performance. Testing the model on diverse datasets, potentially from other domains, would also be valuable to assess its robustness and generalizability. Finally, exploring alternative architectures could provide new solutions to the FP identification problem.

## 5 Conclusions

Our results showed that the Optimized U-Net outperforms the other models, by obtaining the best stability, accuracy, and robustness in learning and predictions. These findings highlighted that the modifications we introduced in the Classic U-Net architecture improved predictions, identifying the Optimized U-Net as the best-performing model. This approach can facilitate large-scale studies investigating the relationship between FP density and taste perception. It could be useful for examining density of papillae in subjects where the coloring with the blue food dye could be difficult to achieve, as in children and young adults who showed higher density of FPs then adults [46], [47]. Besides, it also opens up possibilities for exploring individual differences in taste sensitivity and their links to human health. Furthermore, by making the script available and open source on GitHub, this method can be used by all experts working on taste physiology, and it could also have clinical applications, such as diagnosing taste disorders or monitoring changes in taste sensitivity due to medical treatments or aging. Furthermore, sharing the script on GitHub could also be a fundamental key step for the continuous improvement of the model, which can be achieved through its continuous training with additional datasets.

## Ethics approval and consent to participate

This study was conducted according to the guidelines of the Declaration of Helsinki and approved by the Ethical Committee of the University Hospital of Cagliari (protocol code 451/09, date of approval 5/2016). Informed consent was obtained from all participants enrolled in the study. Study has been registered in www.clinicaltrials.gov, ID number: **UNICADISBMeMe-1.**

## Funding

This research was funded by 10.13039/100014810Fondazione di Sardegna (F73C22001230007 to M.M Convenzione triennale 2021–2023); UniCA - Progetti di Ricerca Start-Up D.M. 737/2021 (F25F21002720001 to M.M. Annualità 2023); POS Italian Health Ministry (F53C22000580001 in 2023).

## Author statement

We confirm that neither the manuscript nor any parts of its content are currently under consideration for publication with or published in another journal. All authors have approved the manuscript and agree with its submission to Computational and Structural Biotechnology Journal.

## CRediT authorship contribution statement

**Melania Melis:** Writing – review & editing, Supervision, Methodology, Investigation, Funding acquisition, Data curation. **Raffaella Fiamma Cabini:** Writing – review & editing, Validation, Software, Methodology, Data curation, Conceptualization. **Diego Ulisse Pizzagalli:** Writing – review & editing, Validation, Supervision, Investigation. **Roberto Crnjar:** Writing – review & editing, Investigation. **Lala Chaimae Naciri:** Writing – original draft, Validation, Software, Methodology, Investigation, Data curation, Conceptualization. **Iole Tomassini Barbarossa:** Writing – original draft, Supervision, Funding acquisition, Conceptualization.

## Declaration of Competing Interest

The authors declare no conflict of interest.

## Data Availability

The analysis code, name and version of the package, and the integrated development environment are available in Github at the link: https://github.com/rcabini/AIpapillae
